# A Case of Hysterical Paraplegia

**Published:** 1867-05

**Authors:** 


*A Case of Hysterical Paraplegia.* Under the care of Dr. Wilks.

Not less interesting, though from a different point of view, is another case in the same ward with the last patient. In this, which is a good specimen of a very common class, the real malady consists in a dormant state of the will. Some interesting remarks on this subject will be found in Dr. Wilks's paper on "Functional Diseases of the Nervous System," in the recent volume of Guy's Hospital Reports. The length of time during which the patient had suffered from her symptoms, and the rapid improvement when she was subjected to the necessary moral treatment, make the case a very striking one. Our account is derived from notes taken by the ward clerk, Mr. J. A. Sharp.

E. C——, aged seventeen, admitted Dec. 3d, 1866. She has always lived at home with her parents, and is the youngest but one of a family of five. Nine years ago she had an attack of what her friends called colic, which lasted two weeks. Five years ago she had rheumatic fever. She had a second attack of colic three years ago, which lasted three weeks. Six months later than this she was taken with pain in the left side and abdomen, which was increased on pressure and on attempting to sit up. After six months there was a remission of the pain, which lasted three months; theni t returned, and has since been increasing and lessening in severity. Two years and five months ago (one month after the commencement of pain in the side and abdomen) she lost the use of her legs, and was unable to sit up; and the pain in the abdomen was increased if she was placed in any but the recumbent posture. The sensation of the legs was not lost, but she occasionally had a kind of twitching and trembling in them. Twelve months ago commenced a frequent barking cough; no expectoration. Six months ago she was mesmerised, and after this it was better for a short time. Her bowels are very costive. When she first took to her bed they were sometimes not open for twenty days; and she now passes seven days without a motion unless purgatives are administered. She has very great pain when a motion is passed. The urine is contained in the bladder at will; and micturition is painful. Has menstruated regularly since thirteen years of age, but the quantity of discharge has always been small. Appetite capricious, but food taken in small quantities is easily digested.

*Condition on admission.*—The appearance of the body generally is healthy and well nourished; the muscles of legs a little wasted; heart-sounds normal; pulse regular, full; lungs resonant; good respiration on both sides; lies constantly on her back in bed, complaining of pain in left side and abdomen; attempts to raise her or sit her up increases the pain; cough frequent, hoarse, barking; no expectoration.

*Progress of case and treatment.*—On Dec. 6th Dr. Wilks ordered her to be slightly faradised, not with the view of its being of any therapeutical value, but simply to occupy the patient's mind with something.

Dec. 8th.—With assistance she got up and sat in a chair sometime without pain. The bowels not having been relieved for several days, Dr. Wilks ordered her one grain each of the extract of nux vomica, extract of aloes, and sulphate of iron, in a pill, to be taken night and morning.

9th.—She again got up for a short time. Cough less frequent and hoarse.

10th.—Bowels relieved for the first time in ten days.

13th.—Bowels again open; cough occasional and has almost entirely lost its hoarse barking character.

14th.—Sits up several hours every day, and by holding on by a chair and pushing it along can walk several yards.

17th.—The bowels are relieved daily. To-day she sat up several hours, and with assistance walked about thirty yards. Has sufficient power in her legs to stand, but in walking requires something to steady her. She still feels pain in her abdomen and side, but it is much less severe than formerly.—*Ib.*

